# Lithographically defined encoded magnetic heterostructures for the targeted screening of kidney cancer†

**DOI:** 10.1039/d3na00701d

**Published:** 2023-12-11

**Authors:** Selma Leulmi Pichot, Tarun Vemulkar, Jeroen Verheyen, Lauren Wallis, James O. Jones, Andrew P. Stewart, Sarah J. Welsh, Grant D. Stewart, Russell P. Cowburn

**Affiliations:** a The Cavendish Laboratory, Department of Physics, University of Cambridge Cambridge CB3 0HE UK; b Semarion Limited CB3 0HE UK; c Department of Surgery, University of Cambridge, Cambridge Biomedical Campus Cambridge CB2 0QQ UK; d Department of Oncology, University of Cambridge, Cambridge Biomedical Campus Cambridge CB2 0QQ UK; e Molecular Immunity Unit, Department of Medicine, University of Cambridge, MRC Laboratory of Molecular Biology Cambridge Biomedical Campus Cambridge CB2 0QQ UK

## Abstract

Renal cell carcinoma (RCC) is the 7th commonest cancer in the UK and the most lethal urological malignancy; 50% of all RCC patients will die from the condition. However, if identified early enough, small RCCs are usually cured by surgery or percutaneous procedures, with 95% 10 year survival. This study describes a newly developed non-invasive urine-based assay for the early detection of RCC. Our approach uses encoded magnetically controllable heterostructures as a substrate for immunoassays. These heterostructures have molecular recognition abilities and embedded patterned codes for a rapid identification of RCC biomarkers. The magnetic heterostructures developed for this study have a magnetic configuration designed for a remote multi axial control of their orientation by external magnetic fields, this control facilitates the code readout when the heterostructures are in liquid. Furthermore, the optical encoding of each set of heterostructures provides a multiplexed analyte capture platform, as different sets of heterostructures, specific to different biomarkers can be mixed together in a patient sample. Our results show a precise magnetic control of the heterostructures with an efficient code readout during liquid immunoassays. The use of functionalised magnetic heterostructures as a substrate for immunoassay is validated for urine specimen spiked with recombinant RCC biomarkers. Initial results of the newly proposed screening method on urine samples from RCC patients, and controls with no renal disorders are presented in this study. Comprehensive optimisation cycles are in progress to validate the robustness of this technology as a novel, non-invasive screening method for RCC.

## Abstract 2

Renal cell carcinoma (RCC) is the 7th commonest cancer in the UK and the most lethal urological malignancy; 50% of all RCC patients will die from the condition.

## Graphical abstract

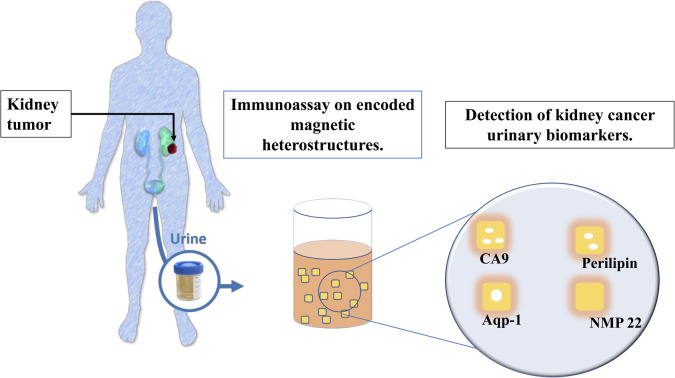

## Introduction

Renal cell carcinoma (RCC; otherwise known as kidney cancer) is the most lethal urological malignancy. There is a great need to further optimise the management of initially localised kidney cancer to improve cure of this lethal cancer.^1^ Kidney cancer is one of few cancers expected to increase in incidence over the next 20 years.^2^

Most people who are diagnosed with kidney cancer do not have any symptoms, including 89% of patients with small renal cancers which are almost always curable by surgery or ablation^3^ (95% 10 year metastasis free survival).^4^ There is an unmet need to develop a screening test for kidney cancer in order to identify asymptomatic disease at an early curable stage.^5^

Urine holds great potential as a non-invasive sampling method for the molecular diagnostic of kidney cancer. Because of the complexity of the tumour biology, one single biomarker is unlikely to be sufficient to establish an accurate diagnostic. Rather, taking into consideration multiple biomarkers levels gives a more accurate diagnostic and hence a better prediction of clinical benefit.^6^

A myriad of urinary protein biomarkers has been studied for the diagnostic of kidney cancer, *e.g.* aquaporin-1, perilipin-2, carbonic anhydrase-9, Raf-kinase inhibitory protein, nuclear matrix protein-22, 14-3-3 protein β/α and neutrophil gelatinase-associated lipocalin.^6^ Despite the multiple studies on these biomarkers, some conflicting results have been reported regarding the biomarkers levels detected in urine from patients with kidney cancer *e.g.* some studies report levels of aquaporin-1 being down regulated in RCC while opposite results are also found in the literature.^6^ Nonetheless, RCC biomarkers are under intense investigation to improve the molecular information needed to tailor the treatment and improve clinical benefit for each patient.

There are currently no screening methods for RCC that could be deployed at large scale in medical settings, although trials are ongoing.^7^ The need for a robust non-invasive screening test motivated the study presented here that evaluates a new immunoassay technique on magnetic heterostructures to screen urine samples for RCC biomarkers.^6^ The urinary immunoassay could be set up for mass screening in a population of individuals identified by clinicians as “at high risk” of developing kidney cancer, such as patients presenting with haematuria for example. The assay described here uses encoded magnetically controllable heterostructures as a substrate for immunoassays. These structures have molecular recognition abilities and patterned codes for rapid analyte identification. Their fabrication process uses techniques from microchip processing such as lithography and physical vapour deposition. A lift off process releases the heterostructures from their substrate into a liquid environment, with their position and orientation in liquid are remotely controlled by externally applied magnetic fields.

**Fig. 1:**
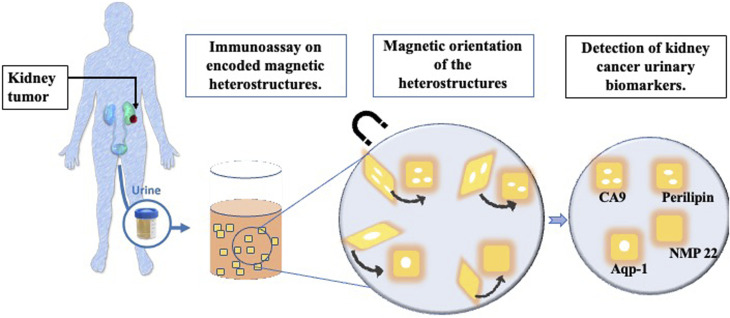
Schematic of the study summary.

The magnetic heterostructures are functionalised with specific capture antibodies which extract a target of interest from the urine samples. Specific detector antibodies highlight the captured target of interest, through the emission of fluorescence. A schematic of the study summary is shown in Fig. 1.

Furthermore, the magnetic heterostructures have a specific topographic component that is used for optical encoding. This feature provides a multiplexed analyte capture platform as each set of heterostructures with a unique code and capture antibody can be mixed together in a patient sample to probe for multiple proteins, in a single experimental run.

The possibility to accurately quantify multiple proteins simultaneously from a single sample is valuable in research and diagnostics. Multiplexed protein level measurements have several benefits, *i.e.*, more data extracted from a minimal sample, reduce the amount of reagents needed, reduced risk of error caused by lengthy procedures, reduced time required for the assay workflow. Existing multiplex immunoassays face the challenge of identifying which analyte is being measured, as the detection method relies widely on the ability to discriminate between emitted wavelengths. Which can be scarce due to the background signal that originates from overlapping excitation emission sources. For this purpose, the newly developed technology on encoded magnetic heterostructures uses a combination of two specific regions; one region that is graphically encoded to create optically detectable features on the heterostructure surface, and a second region for a fluorescence-based analyte detection. The code patterning with photolithography, combined with a detection by the various available fluorophores, offers an immense multiplexing capability.

We investigated the use of such magnetic heterostructures as a substrate for immunoassays for the detection of RCC urinary biomarkers. In an initial set of experiments, phosphate buffer saline (PBS) samples spiked with increasing amounts of human recombinant RCC biomarkers protein (such as AQP-1, perilipin and CA9) were used to validate the ability of the functionalised magnetic structures to accurately detect the levels of spiked proteins in PBS.

In a subsequent set of experiments, taking advantage of the barcode feature on each set of heterostructures, we demonstrated the specificity of the assay. Magnetic heterostructures functionalised to probe for different biomarkers were mixed together in a spiked sample. Only the heterostructures bearing antibodies complementary to the spiked target showed fluorescence.

After validating the robustness of the method in spiked PBS samples, this screening method was deployed for clinical samples. Urine samples from RCC patients, and non-RCC controls, were screened with our new methodology. The data were analysed and compared with results obtained with western blotting. Initial results reveal a high degree of similarity between the two methods. Although further optimization cycles will be required to improve the robustness of the technology, we believe the results presented in this study pave the way for the development of a urine-based multiplex immunoassay for the screening of RCC.

## Methods

### Ethical statement

Urine samples were obtained from patients with RCC using the ethically approved ARTIST (a translational research approach to development of optimal renal cancer treatments in surgical and systemic therapy patients) study. Ethical approval was granted by the East of England, Cambridge South Research Ethics Committee (REC reference 20/EE/0200). Informed consent was obtained for all samples used in this study.

Non-RCC urine samples, referred to in this study as control urine, were provided with the consent of volunteers working in the Cavendish Laboratory. Ethical approval for the collection and use of these samples was granted by the Yorkshire & The Humber – Bradford Leeds Research Ethics Committee (REC reference 19/YH/0125). These individuals have no known current, or history of urological disorders. Informed consent was obtained for all samples used in this study.

An additional control, referred to as control 4 (CTRL 4) consisted in pooled human urine was purchased from Stratech (Cat#IRHUURE-MIN).

### Magnetic heterostructures

**Fig. 2:**
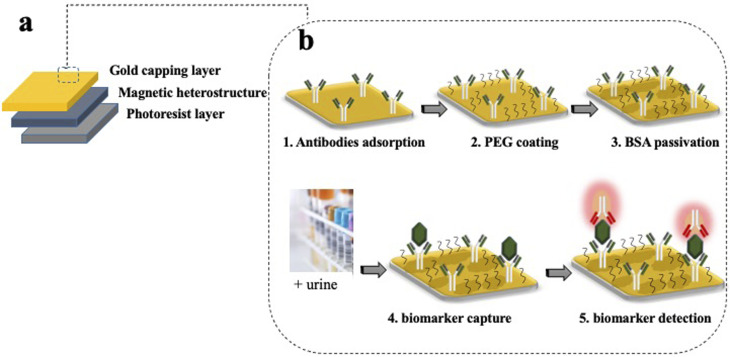
Fabrication of magnetic heterostructures with a molecular recognition ability. (a) The magnetic elements are made from sub-nm alternating layers of CoFeB and Pt in a magnetic heterostructure that is created on a base of a negative photoresist and capped with Au. (b) Schematic of the functionalisation steps on the gold capping layer. (1) Capture antibodies are adsorbed to the gold surface by physisorption. (2) A passivating thiol terminated PEG layer is covalently attached to the gold surface. (3) The heterostructures are incubated with BSA molecules which saturates the sites prone to unspecific bindings. (4) Once incubated with urine, the capture antibodies retrieve the analyte of interest. (5) A fluorescently labelled detection antibody highlights the captured analyte.

Silicon (Si) processing technology and materials from the magnetic memory device industry are used to create magnetically controllable heterostructures. The heterostructures are flat and have optical barcodes to assay more than one biomarker at a time. The heterostructures are fabricated by lithographically patterning 100 μm long and wide, and 1.5 μm thick pillars of a negative photoresist atop a sacrificial layer of dextran on a Si wafer. An array of holes is patterned in each set of structures and serves as a graphical barcode. The patterned pillars are then coated with a magnetic multilayer thin film consisting of alternating, sub-nm thick layers of platinum (Pt) and cobalt–iron–boron (CoFeB) using physical vapour deposition techniques. A capping layer of gold (Au) is then deposited for enhanced fluorescence and allows for surface functionalisation with specific capture antibodies. A schematic of the magnetic heterostructures fabrication process is shown in Fig. 2(a).

The magnetic moment of the heterostructures on the Si wafer is uniformly oriented with a strong uniform magnetic field. Then, the dextran sacrificial layer is dissolved in water, releasing the heterostructures into liquid. For multiplexed assays, each set of uniquely encoded heterostructures is functionalised with capture antibodies that are specific to a unique biomarker. Heterostructures from different sets are then pooled together in suspension to probe for multiple proteins in one patient sample.

An area of magnetic heterostructures attached to the silicon substrate (before the lift off) has been characterized with a Magneto Optical Kerr Effect system (Nanomoke 3, Durham Magneto Optics Ltd). The easy axis hysteresis loop is shown in Fig. 4(b).

To demonstrate that the magnetized heterostructures can be oriented with an external magnetic field, a small volume taken from a suspension of released magnetic heterostructures was dispensed in a 96 well plate and imaged in bright field microscopy. During the imaging, the heterostructures were oriented by an external magnetic field using a neodymium magnet.

### Surface functionalisation

The functionalisation confers to each heterostructure a molecular recognition capability. The gold surface of the magnetic heterostructures is a suitable substrate to immobilize antibodies for immunoassay experiments. Inspired by the well-established capture/detection sandwich immunoassays, the magnetic heterostructures are coated with specific capture antibodies that pull the biomarker of interest from the complex urine medium. A complementary specific detection antibody highlights the captured target of interest, through the emission of fluorescence.

The functionalisation starts by cleaning the magnetic heterostructures in a solution of 30% H_2_O_2_ to remove organic contaminants from their gold surface. After a thorough rinse in ultra pure water, the heterostructures are incubated in a PBS solution containing 1 μg mL^−1^ of specific capture antibodies. The antibodies directly adsorb to the gold layer through a physisorption process. After successive washes to remove unbound antibodies, the antibody coated structures are incubated in a thiolated polyethylene glycol (PEG) solution (3.6% in PBS) (mPEG Thiol 11156-0695, Polypure). The short PEG molecules bind to available areas of the gold surface through the thiolated group, the passivating effect of the PEG layer reduces the tendency of the proteins to bind to the heterostructure's surface through non specific interactions. Finally, after successive washes to remove unbound PEG molecules, the magnetic heterostructures are incubated in a 0.5% bovine serum albumin (BSA) solution that saturates the sites prone to unspecific protein adsorption. A schematic of the functionalisation strategy is shown in Fig. 2(b).

### Urine analysis

Following informed consent, pre-operative urine samples were collected from 8 patients with pathologically confirmed renal carcinoma. Control non-RCC urine samples were collected from 3 healthy volunteers. Urine was taken and stored at −80 °C until use.

Untreated urine (urine non treated with ethylenediaminetetraacetic acid (EDTA)) was used for all experiments, with samples mixed thoroughly prior to use to resuspend pelleted and aggregated materials.

### Western blot screening

Selected urine samples were screened for biomarkers using a sensitive and specific western blot (WB) technique. Urine samples were diluted in lithium dodecyl sulfate (LDS) sample buffer and a reducing agent (both reagents from NuPage™, Invitrogen™) and heated during 10 minutes at 70 °C to ensure the denaturation of the soluble proteins in urine. 30 μL of each sample was loaded in the gel for the electrophoresis.

After transfer, membranes were blocked overnight in a blocking buffer containing 0.1% Tween-20. The membranes were then incubated with anti-AQP1 (Ab9566, Abcam) anti-CA9 (ab15086, Abcam) or anti-ADFP antibody (PA5-29099, Invitrogen™) all at a concentration of 1 μg mL^−1^ for two hours. After washings, the membranes were incubated with a 1 : 1000 dilution of goat anti-rabbit IgG conjugated with horseradish peroxidase (G-21234, Invitrogen) for one hour. All antibodies incubation were done in blocking buffer with 0.1% Tween-20.

Urinary AQP1, CA9 and perilipin were visualized and quantified (in arbitrary chemiluminescence units) using an infrared imager (GelDoc-It™).

### Biomarkers screening with encoded magnetic heterostructures

Urine samples from patients and controls screened with WB were analysed subsequently with the newly developed immunoassay on magnetic heterostructures. The aim is to assess the robustness of this method compared to WB. The functionalised magnetic heterostructures were dispensed in urine samples from each participant. The reaction was left to incubate for 2 hours at room temperature, or overnight at 4 °C to promote the binding of urinary AQP1, CA9 and PLIN to the capture antibodies immobilized on the heterostructures surface. After the incubation time, the urine samples were discarded, and the heterostructures washed with PBS containing 0.01% Tween-20. Between washes, the heterostructures were allowed to settle for 1 min and then the liquid was removed with a manual pipette.

The magnetic heterostructures are then resuspended in a solution of fluorescently labelled detection antibodies for either AQP1 (ab225225, Abcam), CA9 (ab225074, Abcam) or perilipin (ab201535, Abcam) at a concentration of 1 μg mL^−1^ and left to incubate for 1 hour. Several washes are finally performed to remove unbound fluorescent antibodies. During the assay, neodymium magnets are used to keep the heterostructure gold face-up when during the incubation times, and to align them (face-down) with the microscope objective prior to imaging.

### Multiplex experiment

**Fig. 3:**
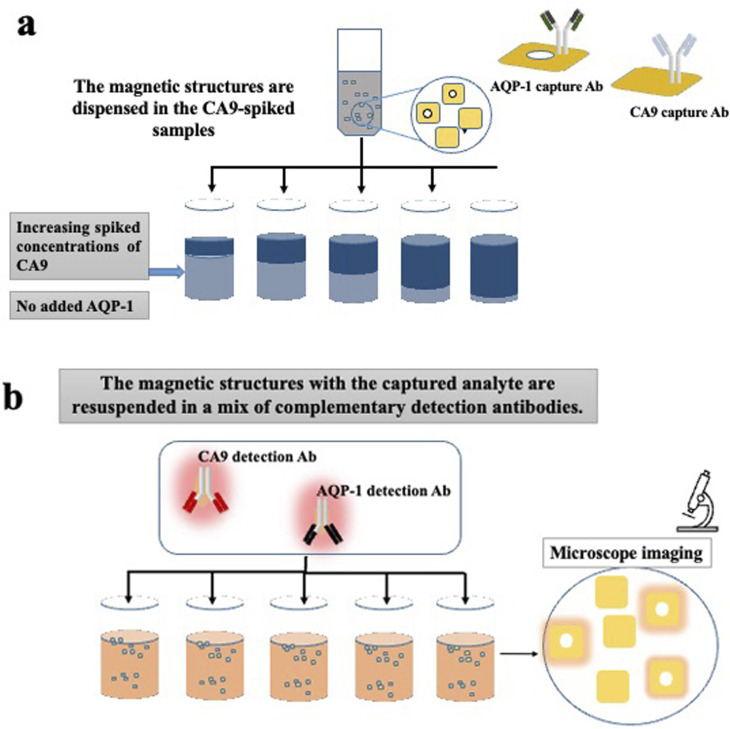
Illustration of the multiplex assay procedure. (a) Two sets of barcoded heterostructures functionalised with capture antibodies, specific to CA9 or AQP-1, are dispensed in PBS spiked with increasing concentrations of human recombinant CA9. (b) The heterostructures are retrieved and resuspended in a mixture of two, fluorescently labelled, detection antibodies (anti CA9 and anti AQP-1). In the final step, the heterostructures are oriented with a magnetic field to align their surface for imaging. The barcode reading assigns the fluorescence intensity to a specific known biomarker.

For the multiplex experiments, PBS was used as a medium. PBS is divided into multiple aliquots and then spiked with commercial recombinant proteins. In the first set of experiments, PBS aliquots were spiked with increasing concentrations of recombinant human CA9. No aquaporin was added in none of the samples. Then two separate sets of barcoded heterostructures were added to the sample; the first set with barcode 1 (plain) were functionalised with CA9 capture antibodies, the seconds set with barcode 2 (with hole) were functionalised with AQP1 capture antibodies. The samples were left to incubate a sufficient time for the analytes capture. After multiple washing steps, the functionalised magnetic heterostructures were dispensed in a suspension containing a mix of complementary detection antibodies, fluorescently labelled; anti-CA9 and anti-AQP-1. The experiment is detailed in Fig. 3.

## Results

### Magnetic heterostructures

**Fig. 4:**
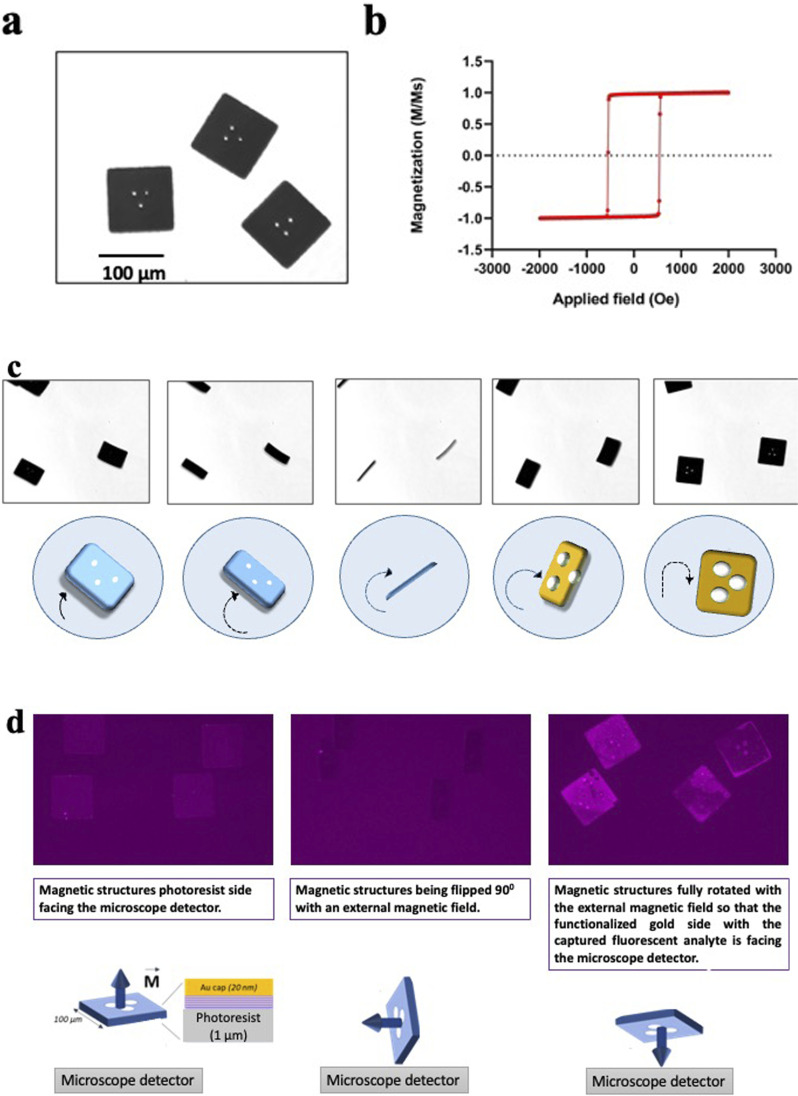
Magnetic properties of the heterostructures. (a) Released flat heterostructures imaged in liquid with bright field microscopy. (b) Hysteresis loop representative of the magnetic stack embedded in the heterostructures. (c) Magnetic orientation of the heterostructures. Sequence of microscope images showing the movement of the heterostructures while being subjected to an external moving magnetic field. Heterostructures can be controlled in plane and out of the plane. The encoded pattern becomes clearly visible again when the heterostructures land on the well plate. (d) Orientation with a magnet in fluorescence microscopy. The first image (from the right) shows the heterostructures with the resist side facing the microscope detector. The second image is taken with the magnet flipping the heterostructures on their side. The last image shows the heterostructures fully flipped (180°) with their functionalised gold side facing the microscope detector. The fluorescence on the heterostructures is originated from a fluorescently labelled secondary antibody bound to a captured analyte (human recombinant AQP-1).

The microfabrication process has been tailored to make structures that are effectively flat as seen in Fig. 4(a). The flat surface is important during the imaging process; it allows an accurate barcode readout in bright field microscopy, with a maximized signal detection during fluorescence imaging.

The magnetic stack is characterized by strong out-of-plane magnetic anisotropy, interlayer dipole coupling, and sharp, high coercivity magnetic switching as seen in Fig. 4(b) These properties provide each heterostructure with a high magnetic moment that points out of its plane, perpendicular to the surface normal. This property allows for a multi axis manipulation of the heterostructures in liquid with remote magnetic fields, as shown in Fig. 4(d).

Fig. 4(c) shows a sequence of microscope images of the heterostructures while a neodymium magnet is moved closed by. The heterostructures follow precisely the movement of the external magnetic field, the magnetic torque exerted on the heterostructures with the perpendicular magnetic anisotropy flip each heterostructure out of the well plate plane with a precise angle determined by the user. The magnetic heterostructures can also be controlled in plane and are observed to follow the same movement direction as the magnet (compare images 2 and 3).

Despite a high aspect ratio and the nanometric thickness of the magnetic multilayer stack, the sheet-like structure of each magnetic heterostructure exhibit a high structural stability. Indeed, the heterostructures do not bend or show any deformation while the fluid-generated strain is perpendicular to the plane of the heterostructure surface during the flipping process. The encoded pattern becomes clearly visible again as soon as the heterostructures land flat back again on the well plate, as seen on the Fig. 4(c).

In the immunoassay for the RCC biomarkers detection, the magnetic spatial control orients the gold surface of the heterostructures, which ensures that the functionalised side with captured biomarkers is always aligned with a detector or imaging device. Fig. 4(d) shows how the heterostructures orientation is controlled remotely with a neodymium magnet during the immunoassay. The heterostructures are first imaged with the negative resist side facing the detector, then the magnet rotates the heterostructures 90° perpendicularly to the well plate plane, and finally, the heterostructures are fully flipped so that the gold covered side with the detected analyte (human recombinant AQP-1 on Fig. 4(c)) is now facing the detector.

### Immunoassay on encoded magnetic heterostructures

**Fig. 5:**
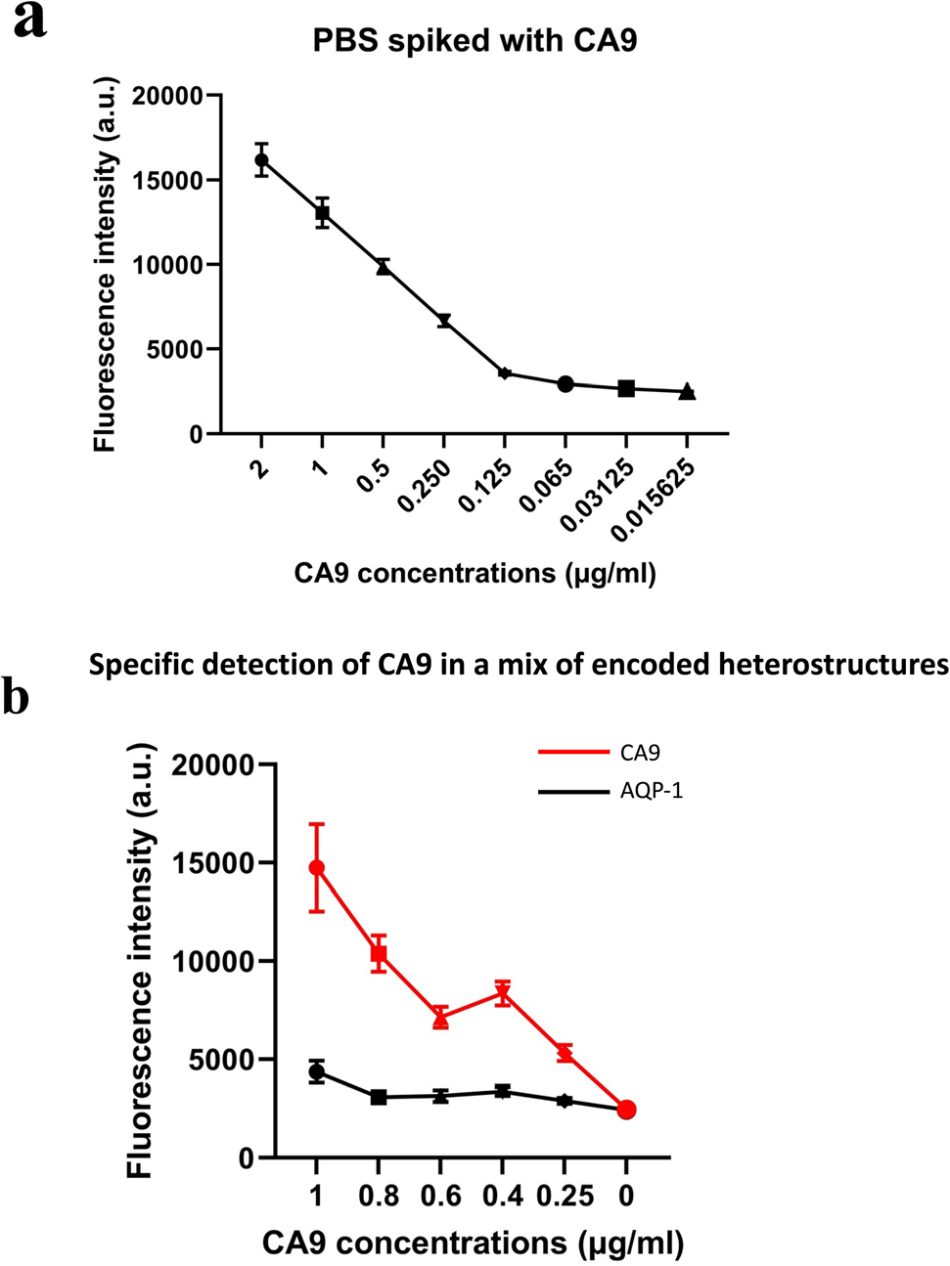
(a) Fluorescence intensities of functionalised magnetic heterostructures in PBS samples spiked with human recombinant CA9. (b) Fluorescence intensities of two sets of functionalised magnetic heterostructures. The fluorescence is measured in PBS spiked with increasing concentrations of human recombinant CA9. *n* ∼ 100.

Initial results on spiked PBS samples show that the fluorescence quantification on the functionalised magnetic heterostructures reflected accurately the concentrations of spiked human recombinant biomarkers. The detected fluorescent signal was proportional to the CA9 concentration as shown in Fig. 5(a). Similar results were observed with other spiked RCC biomarkers; NMP22, AQP-1 and perilipin (data not shown).

These results also highlight that the functionalisation process was effective in capturing the soluble biomarkers in the samples. The fluorescence is quantified in arbitrary fluorescence units with ImageJ software.

### Multiplexed immunoassay on encoded magnetic heterostructures

The multiplex experiments results were in line with the designed experiment. An ImageJ script was developed to decode each set of heterostructures when mixed together in a sample and measures the fluorescence intensity (in arbitrary units) in each subpopulation of magnetic structures. The fluorescence signal retrieved from the set of anti CA9 heterostructures (with barcode 1) shows an increase in signal intensity proportional to the spiked concentration, while the analysis of anti AQP1 heterostructures (with barcode 2) was low and constant across all the samples tested as shown in Fig. 5(b). Error bars represent standard error of the mean of the data set (fluorescence intensity on each individual heterostructure).

### Clinical urine analysis

**Fig. 6:**
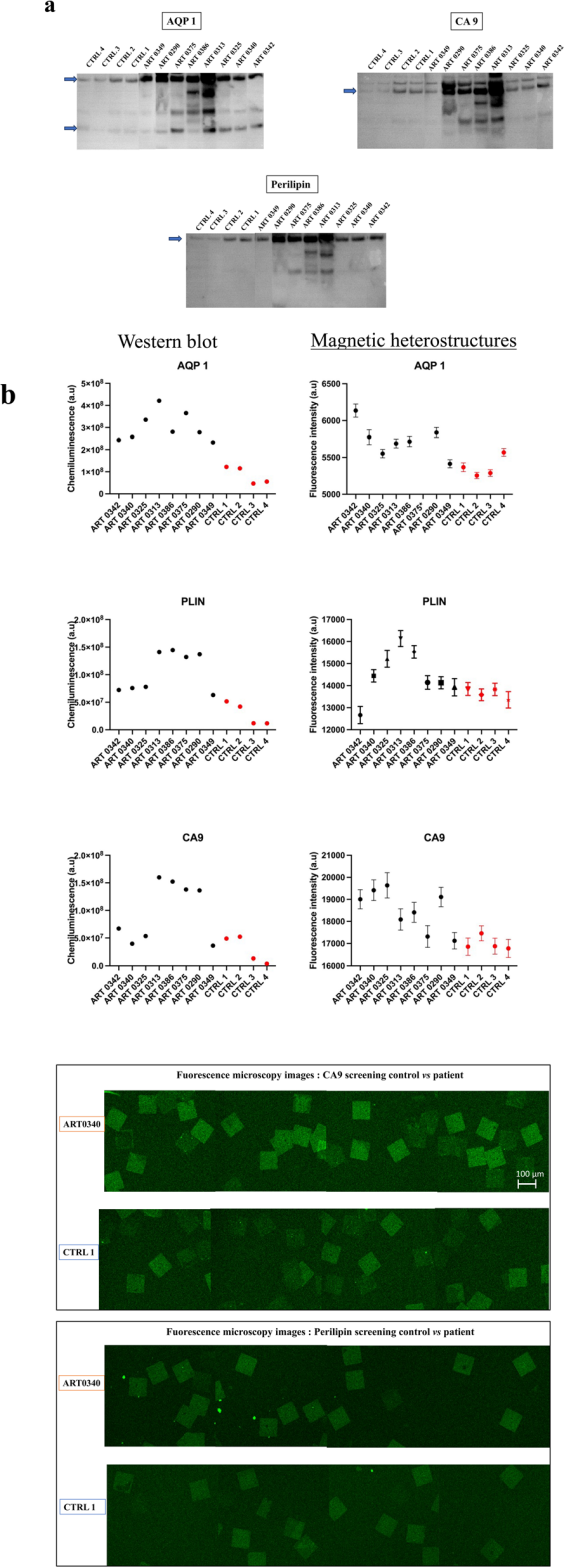
Urine analysis. Samples from patients with diagnosed RCC, and from non-RCC control individuals were screened for AQP1, perilipin (PLIN) and CA9. (a) Quantification of the bands intensities on the western blot membranes (arrows point to the measured band). For AQP1, the western blot revealed two bands, a lower 28 kDa band corresponding to unglycosylated form and an upper high molecular ∼50 kDa glycosylated form. For each individual, the sum of the two bands intensities is plotted on the graph (b) the immunoassay on magnetic heterostructures. Data shown on the graph represent the mean of ∼100 measurements per urine sample (*n* ∼ 100 heterostructures analysed per sample). Error bars represent the standard error of the mean. *Data point outside the axis limit. (c) Fluorescence microscopy images of functionalised magnetic heterostructures after the urinary screening for CA9 and perilipin. Snapshots from two sets of samples have been taken for comparison; control 1 (CTRL 1) and the patient sample ART0340.

Urine from patients with RCC and from non-RCC control individuals were analysed with western blot and screened for CA9, AQP1 and perilipin. Details of the tumour size and subtype for each patient are shown in Table 1. Results reveal that urine samples from some patients with RCC exhibited higher urinary concentrations in CA9, AQP1 and perilipin compared to controls as shown in Fig. 6(a).

**Table 1:** Tumour sizes for patients with confirmed RCC whose urine specimens were analysed with WB and the immunoassay on magnetic heterostructures

ARTIST ID	ART 0342	ART 0340	ART 0325	ART 0313	ART 0386	ART 0375	ART 0290	ART 0349
Tumour size	6 cm	9.2 cm	6.5 cm	9 cm	10 cm	5 cm	6 cm	4.6 cm
Subtype	ccRCC	ccRCC	ccRCC	ccRCC	ccRCC	ccRCC	ccRCC	ccRCC

Urine samples screened subsequently with the functionalised magnetic heterostructures exhibited a similar trend as WB, with higher concentrations of CA9, AQP1 and perilipin for some patients with known RCC than in control subjects, as shown in Fig. 6(b). Around 100 heterostructures per sample were used for the immunoassay, the fluorescence of each heterostructure was individually quantified. The data points on the graph show the mean fluorescence of ∼100 measurements per sample (*n* ∼ 100). While the chosen sample size may appear arbitrary to some readers, its selection was purposeful within the context of this study. We aimed to strike a balance between ensuring sufficient statistical power to detect meaningful effects on the heterostructures and the practical constraints of data collection within our experimental resources.

Specifically, the immunoassay on the magnetic heterostructures was conducted using a 96-well plate, although this method isn't mandatory and can be performed on various substrates. Through multiple imaging sessions, we determined that an approximate density of 100 heterostructures per well was optimal for achieving individualized heterostructures during fluorescence imaging. A higher particle density would lead to encumbrance within each well, resulting in overlapping structures that hinder accurate image analysis. A significant difference in biomarkers levels was found between the samples (one-way Anova *p* < 0.0001 for all the biomarkers tested). A *post hoc* Dunnett test confirmed that the levels of CA9 and perilipin were significantly higher in the urine from some patients compared to the control individuals. For AQP1, all the patients tested exhibited higher levels that the controls. Fig. 6(c) shows fluorescence microscopy images of the functionalised heterostructures after the immunoassay. Snapshots taken from control 1 (CTRL 1) and the patient sample ART0340 are shown to illustrate the corresponding readings reported on Fig. 6(b). A summary of the statistical analysis between the patients *vs.* controls urine samples is shown in Table 2. This observation highlights the need to evaluate multiple proteins because the pattern of their urinary levels varies from patient to patient. One may notice that the biomarkers semi-quantitative profiles exhibit some dissimilarities. This is due to the difference between the two detection methods namely chemiluminescence for WB and direct fluorescence for the immunoassay on particles. Chemiluminescence provides an indirect assessment of protein quantities. The detected light signal does not linearly correlate with protein concentrations. This is due to the exponential signal amplification through the enhanced chemiluminescence (ECL) substrate interaction with horseradish peroxidase (HRP) conjugated antibodies. On the contrary, the fluorescence signal provides a more direct reading as the detection antibodies are directly labelled to the fluorophores. Overall, the two methods were consistent in showing that urinary CA9, AQP1 and perilipin levels were higher in patients with confirmed RCC than in control individuals.

**Table 2:** Dunnett statistical analysis for the comparison of biomarkers levels, between patients and controls, measured with the immunoassay on magnetic heterostructures. *p < 0.05, **p < 0.01, ***p < 0.001, ****p < 0.0001, ns: non-significant. Colour code: top red is for CA9, middle black is for PLIN, bottom green is for AQP 1

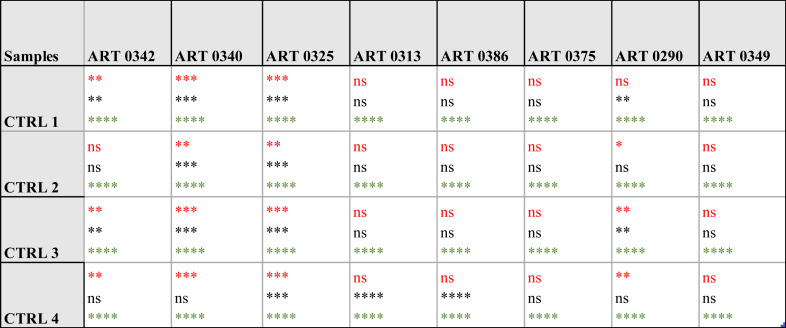

## Discussion

Data presented in this study confirm the feasibility of the newly designed immunoassay platform for the detection of urinary biomarkers for renal cancers diagnosis, for which there are currently no population level screening methods available. This study brings together expertise from clinical medicine, material science, surface chemistry and molecular biology to develop a technology that discriminates between individuals with or without RCC.

The design of controllable, encoded magnetic heterostructures as a substrate for the well-established capture/detection sandwich immunoassays provides a new insight in the field of diagnostic. A new class of magnetic anisotropic structures that can be remotely actuated have been previously described and their use is actively investigated in biomedical research^8–10^ In this study, the barcode on each heterostructure gives a distinct optical reading, which, when combined to fluorescence detection gives precise information about which biomarker is being measured. The patterning with photolithography offers a high coding capacity that would allow to probe for thousands of molecules in a unique sample. Simultaneous barcodes readout of hundreds of heterostructures is facilitated by the magnetic positioning prior to the imaging, the heterostructures are then easily identifiable with bright field microscopy.

The barcode decoding process is very simple, it relies on the image contrast generated by the heterostructures with the transmitted light. In this study, we used the open source image analysis software (Fiji)^11^ for the decoding process. A major advantage of this readout method is the use of one single tool for both graphical decoding and fluorescence analysis simultaneously. Another advantage is that Fiji is already widely used by biologists for fluorescence analysis in bioassays.

A plethora of barcoded particles technologies for multiplexed assays have been developed in the recent years. Fluorescence based barcode particles are the most widely used and are already commercially available such as the Luminex xMap technology. Various other encoding strategies have also been proposed such as graphic encoding^12–17^ magnetic encoding^18–20^ and electrical encoding.^21^ While development of such technologies holds great promises, a sophisticated equipment for the signal detection and analysis could be challenging to implement for routine screening in laboratories with standard molecular biology equipment. This issue raises concerns about the transferability and reproducibility of results between research groups. The approach described in this study has the advantage of using benchtop microscopes widely available in most biological and medical laboratories. Indeed, the optical barcode is read with the transmitted light in bright field mode, the fluorescence detection is achieved with a wide selection available lasers or filter cubes.

While the graphical binary encoding offers an easy optical readout with many possible encoding combinations, one could argue that the number of distinguishable patterns with actual microscopes is limited by the heterostructure surface area. To overcome this limitation, this study demonstrates the combination of surface pattern encoding with the fluorescence encoding, widely used in the molecular biology community, combining advantages from both approaches for the multiplexed analyte detection.

A multiplexed evaluation of multiple biomarkers is of a great importance to guide the clinical treatment plan for each patient. In the context of kidney cancer, a panel of urinary protein biomarker that can help an accurate diagnosis of renal masses have been reported in the literature. While urine represents an ideal source for a non-invasive sampling, working with urine in this study posed significant challenges, especially to detect low abundant biomarkers. Urine contains more than 1500 proteins, the majority of which are membrane bound.^22^ Urine also contains organic molecules, such as urea and creatinine, and cellular debris. All these substances hinder the efficient binding of the biomarkers to their corresponding capture antibody in immunoassays. Previous work has suggested that diluting the urine sample led to an attenuation of the matrix effect and led to an improved protein quantification.^23^ However, the urine sample dilution was effective only when endogenous protein concentrations were well above the limit of quantification but was ineffective when concentrations were close to the limit of quantification of the assay. In our set of experiments, various dilutions conditions have been tested including different dilution ratios and the use of different diluents with and without detergents. However, the dilution did not show a noticeable improvement in detecting RCC biomarkers in patients' urine.

Urine specimens have a high degree of variability due to age, health, diet and pH. Another important parameter is the proteolysis that might occur while the urine is stored in the bladder. Such variations are significant whether they are between individuals, or within each individual.^23,24^ Further, a lack of standardized protocols in urine analysis has led to varying and sometimes conflicting results.^25^

Our data did not show a relation between the tumour size and the AQP-1 or perilipin levels as previously reported by Morrissey *et al.*^26^ even after normalizing the results to urine creatinine following a protocol similar to the one reported by Morrissey *et al.* A possible reason being the complication to replicate the experiment realized by Morrissey's group due to discontinued commercial antibodies and use of proprietary antibodies. However, our results, although on a small number of participants, are in line with Morrissey's results in showing that, some patients with RCC, do exhibit higher levels of certain urinary biomarkers than controls with no known history or current renal disorders.

Some urine samples from patients with RCC did not have concentrations higher than those of the controls. Suggesting that the biomarkers concentrations were very low, near or below the limit of detection of the assays. Another explanation would be the freezing process of the urine samples which might have led to protein degradation. Indeed, in the work realised by Sreedharan *et al.*^27^ the WB analysis of RCC patient urine showed no signal for archived samples. On the contrary, the same analysis on fresh urine samples from RCC patients exhibited elevated levels of AQP and perilipin compared to control subjects. Kidney cancers are also genetically heterogeneous, as a result, the levels of urinary biomarkers vary for different patients and for each protein. Therefore, one key to an optimised treatment is to map the profile of multiple urinary biomarkers for each patient. In his work, Morrissey *et al.*^26^ reported that assessing two biomarkers AQP-1 and perilipin-2 demonstrated that patients with kidney cancer had elevated levels these two biomarkers compared to control individuals. In a subsequent study,^28^ the authors developed an algorithm to analyse the aquaporin 1 and perilipin 2 levels in conjunction with renal mass biopsy. Their results suggest that aquaporin 1 and perilipin 2 possess high sensitivity and specificity for detecting clear cell and papillary renal cell carcinoma. Although research in this area is still limited and would benefit from concomitant results from several research groups, such combinatorial approaches highlight the need for a rapid and specific multiplexed assay as a clinical decision-making support for the individualized treatment of kidney cancer patients.

To conclude, this study demonstrates the development of functional encoded magnetic heterostructures. The heterostructures have a perpendicular magnetic anisotropy that enables a multiaxial control of their positioning in fluids with remote magnetic fields. This study explored the use of such heterostructures for immunoassays, and their use to screen individuals with a confirmed ccRCC. Although our investigation is limited by a small number of participants, the initial results are encouraging. A further study, which will be a clinical validation study, is under consideration where the newly developed technology is utilised across a larger cohort of patients with RCC.

## Conflicts of interest

Grant D. Stewart has received educational grants from Pfizer, AstraZeneca and Intuitive Surgical; consultancy fees from Pfizer, Merck, EUSA Pharma and CMR Surgical; travel expenses from Pfizer and Speaker fees from Pfizer. Jeroen Verheyen and Tarun Vemulkar are employees of Semarion. Russell P. Cowburn is non-executive director of Semarion. Russell P. Cowburn is employed by Durham Magneto Optics. All other authors have no financial disclosures.

## Supplementary Material

NA-006-D3NA00701D-s001

NA-006-D3NA00701D-s002
