# Supplementary material for: Lithographically defined encoded magnetic heterostructures for the targeted screening of kidney cancer
Source: Nanoscale Adv. 2023 Dec 11;6(1):276–86. doi: 10.1039/d3na00701d (PMC10729922; doi:10.1039/d3na00701d)
Supplement: NA-006-D3NA00701D-s001 [file NA-006-D3NA00701D-s001.pdf]

Electronic Supplementary Material (ESI) for Nanoscale Advances.
This journal is © The Royal Society of Chemistry 2023

Supplementary information

Below is a comparative table which summarises the key experimental requirements such as the sample volume and the reaction time required in conventional methods such as ELISA and the new method (Immunoassay on magnetic heterostructures).

Conventional Elisa			
	Sample volume	washing steps	Reaction time
CA9	100 µl	8	4.5 Hours
AQP-1	100 µl	8	4.5 Hours
PLIP	100 µl	8	4.5 Hours
NMP22	100 µl	8	4.5 Hours
**Total for the screening of 4 biomarkers**	**400 µl**	**32 washing steps**	**18 Hours ***

immunoassay on encoded magnetic heterostructures			
	Sample volume	washing steps	Reaction time
CA9			
AQP-1			
PLIP			
NMP22	100 µl	10	4 Hours
**Total for the screening of 4 biomarkers**	**100 µl**	**10 washing steps**	**4 Hours**

Figure. Comparison between Conventional ELISA and the immunoassay on magnetic heterostructures. The kit used for the comparison is the Human Carbonic Anhydrase IX Quantikine ELISA Kit (R&D Systems Catalog #: DCA900), which is used for measuring human CA9 in urine samples. (*) The reaction time to screen for the four biomarkers could be reduced if the samples are run in parallel, however, this might introduce a higher risk of experimental errors due to a higher level of manual handling.
