# Supplementary figures and images for: Lithographically defined encoded magnetic heterostructures for the targeted screening of kidney cancer
Source: Nanoscale Adv. 2023 Dec 11;6(1):276–86. doi: 10.1039/d3na00701d (PMC10729922; doi:10.1039/d3na00701d)

AQP-1

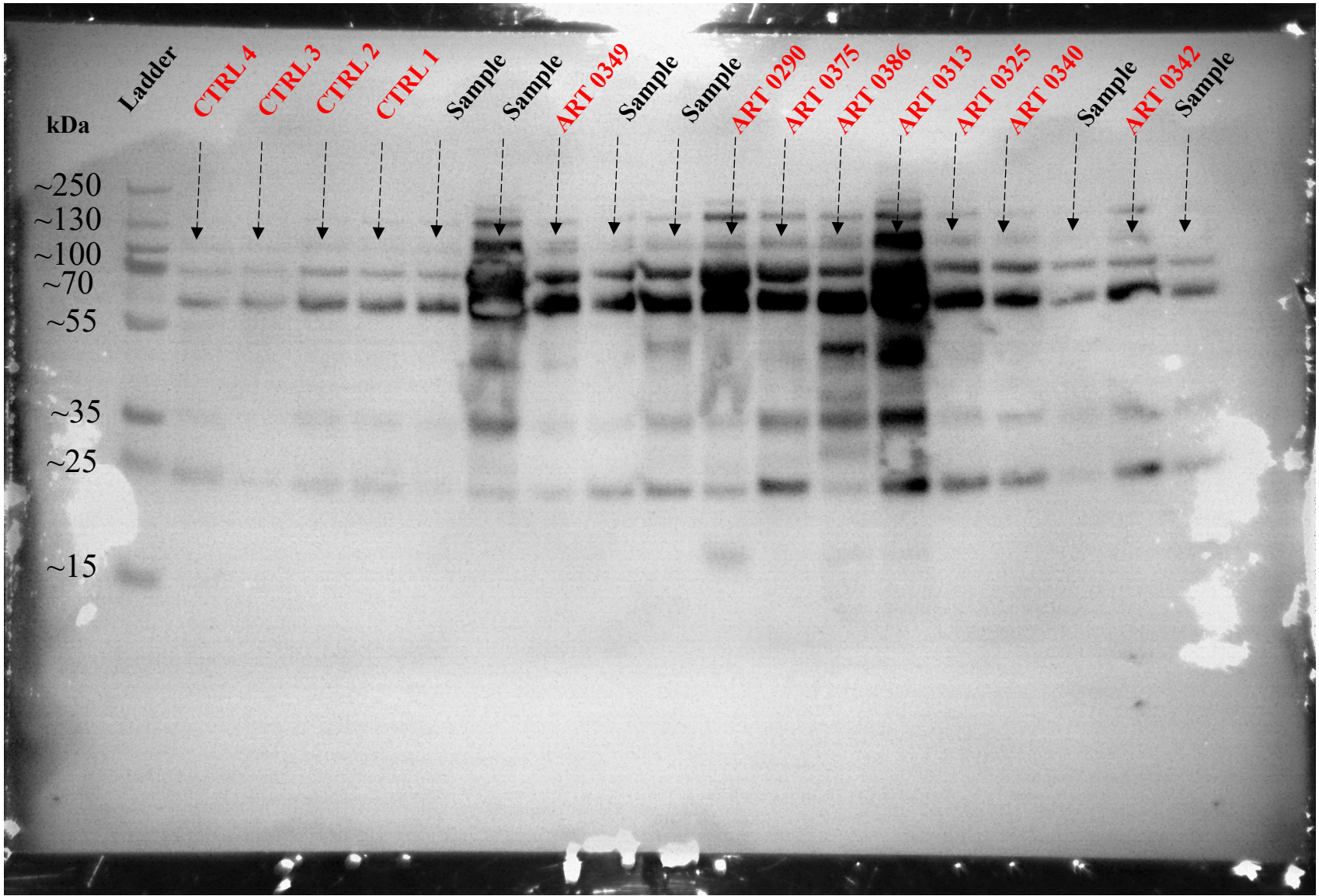

# CA-9

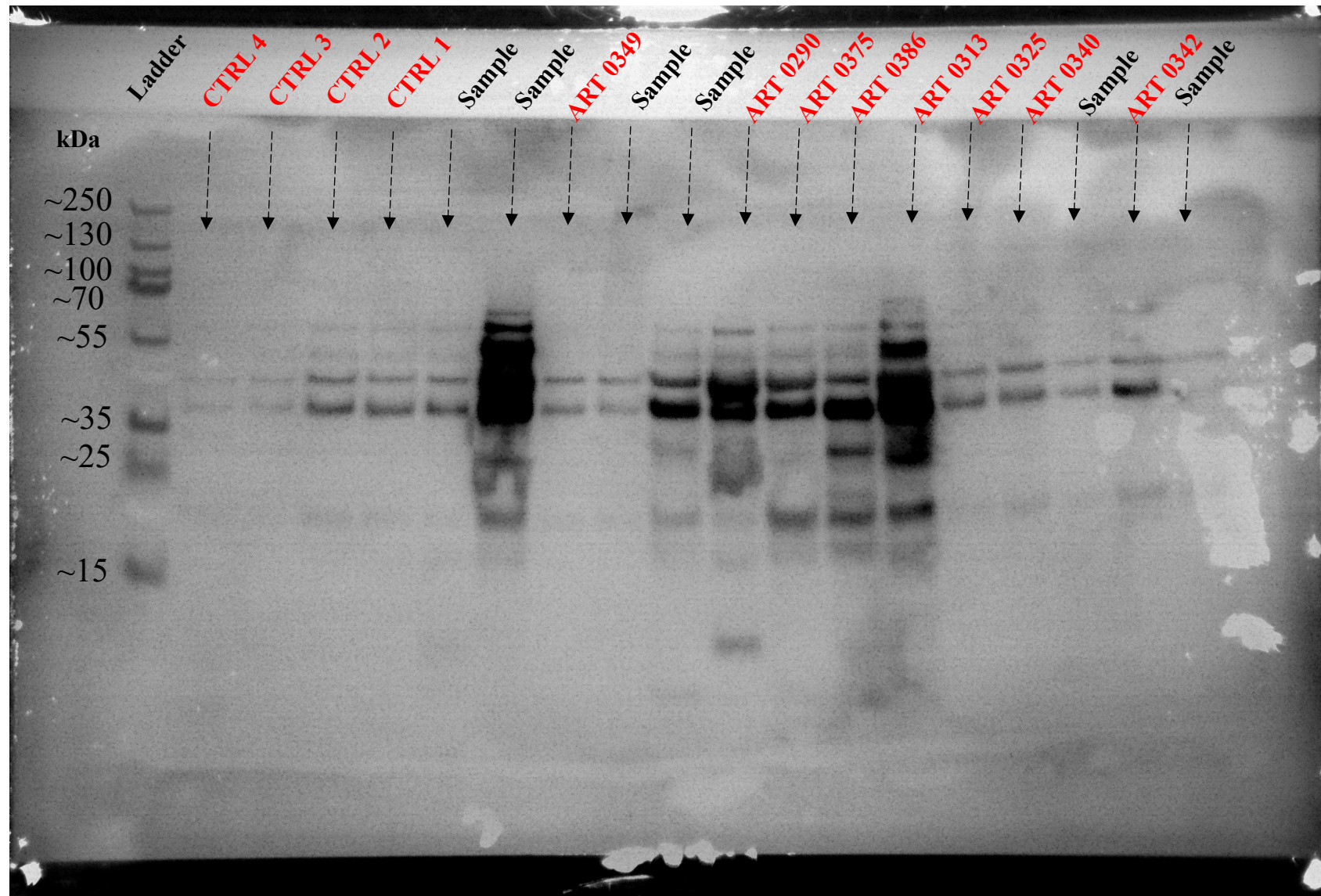

# Perilipin

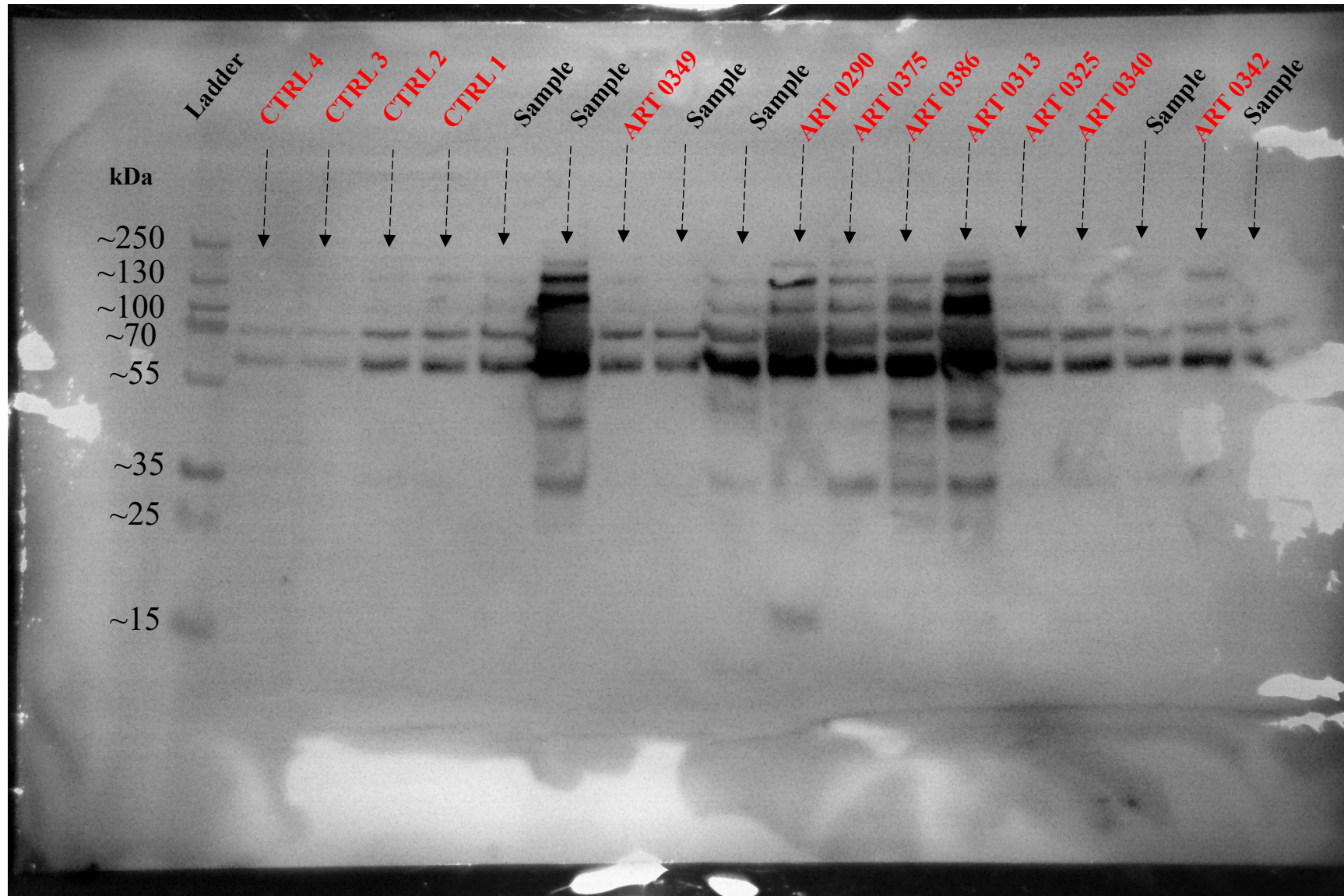

Supplement: NA-006-D3NA00701D-s002 [file NA-006-D3NA00701D-s002.pdf]
